# Impact of modeled microgravity stress on innate immunity in a beneficial animal-microbe symbiosis

**DOI:** 10.1038/s41598-024-53477-3

**Published:** 2024-02-05

**Authors:** Alexandrea A. Duscher, Madeline M. Vroom, Jamie S. Foster

**Affiliations:** 1 Department of Microbiology and Cell Science, Space Life Science Lab, University of Florida, Merritt Island, FL 32953 USA; 2 Present Address: Chesapeake Bay Governor’s School, Warsaw, VA 22572 USA; 3 Present Address: Vaxxinity, Space Life Sciences Lab, Merritt Island, FL 32953 USA

**Keywords:** Immunology, Microbiology

## Abstract

The innate immune response is the first line of defense for all animals to not only detect invading microbes and toxins but also sense and interface with the environment. One such environment that can significantly affect innate immunity is spaceflight. In this study, we explored the impact of microgravity stress on key elements of the NFκB innate immune pathway. The symbiosis between the bobtail squid *Euprymna scolopes* and its beneficial symbiont *Vibrio fischeri* was used as a model system under a simulated microgravity environment. The expression of genes associated with the NFκB pathway was monitored over time as the symbiosis progressed. Results revealed that although the onset of the symbiosis was the major driver in the differential expression of NFκB signaling, the stress of simulated low-shear microgravity also caused a dysregulation of expression. Several genes were expressed at earlier time points suggesting that elements of the *E. scolopes* NFκB pathway are stress-inducible, whereas expression of other pathway components was delayed. The results provide new insights into the role of NFκB signaling in the squid-vibrio symbiosis, and how the stress of microgravity negatively impacts the host immune response. Together, these results provide a foundation to develop mitigation strategies to maintain host-microbe homeostasis during spaceflight.

## Introduction

Spaceflight-induced dysregulation of the innate immune system has long been recognized as a key health issue for astronauts working in the space environment^1–3^. Since the time of the first manned flights, more than 50% of returning astronauts have shown evidence of impaired immune function^4–6^. Although there are many different environmental hazards associated with spaceflight that may contribute to the altered immune response, microgravity has been shown to impair innate immune cell differentiation, function, and signal transduction^6–10^. For example, analysis of monocytes collected from nine astronauts after a two-week spaceflight showed impaired cell adhesion, migration, and cytokine secretion after stimulation with microbial-associated molecular patterns (MAMPs)^11^.

One key pathway of the innate immune system thought to be associated with the immune dysregulation phenomena in microgravity is the NFκB signaling pathway^12–15^. The NFκB signaling pathway is found in most, if not all, animals and is comprised of transcription factors that are retained in the cytoplasm of a cell until upstream signaling events trigger translocation of homo- or heterodimers into the nucleus where they initiate transcription of NFκB-controlled innate immune genes^16^. NFκB signaling can be initiated and regulated through the recognition of MAMPs, as well as by reactive oxygen species (ROS)^17,18^. During spaceflight, gene expression of the NFκB pathway is disrupted in a wide range of cell types^6,15,19,20^ that may impact bone and muscle loss in vertebrates, as well as regulation of key immune and inflammatory responses^21,22^.

**Figure 1:**
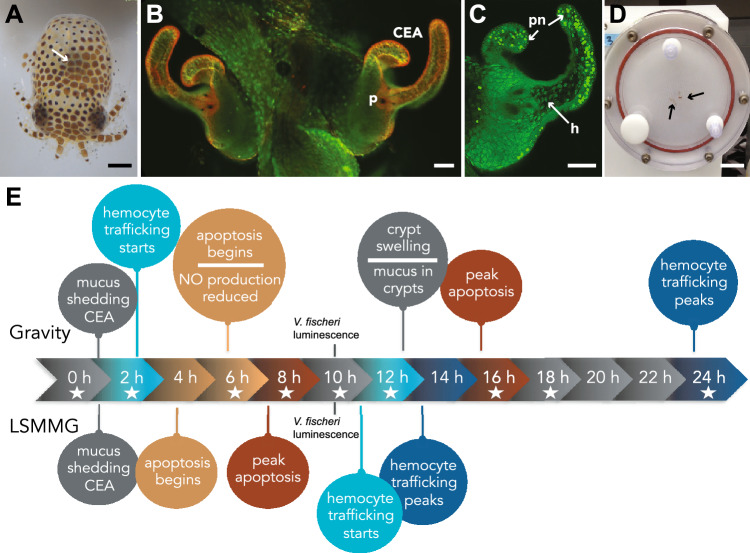
Overview of the *Euprymna scolopes* and *Vibrio fischeri* symbiosis. (**A**) Hatchling *E. scolopes* with the location of the symbiotic light organ depicted by the white arrow. Bar, 500 µm. (**B**) Epifluorescent image of the nascent light organ stained with acridine orange visualizing the superficial ciliated epithelial appendages (CEA) that entrain bacteria into the vicinity of pores (p) on the surface of the light organ. Bar, 50 µm. (**C**) One-half of the light organ 16 h after inoculation with symbiosis-competent strains of *V. fischeri*. Acridine orange staining reveals the pattern of pycnotic nuclei (pn) typically associated with the bacteria-induced apoptotic cell death event. The symbiotic *V. fischeri* also triggers the migration of macrophage-like hemocytes (h) into the blood sinus underlying the CEA. Bar, 50 µm. (**D**) Example of a high aspect-ratio vessel (HARV) used to simulate low-shear modeled microgravity (LSMMG). Squid, indicated by the black arrows, are in “free fall” suspended in the middle of the HARV. Bar, 3 cm. (**E**) Timeline of bacteria-induced developmental events in the host under normal gravity conditions compared to low shear modeled microgravity conditions (LSMMG). Stars indicate time points targeted for NanoString gene expression assay.

To further explore how the NFκB signaling pathway is impacted by spaceflight-like conditions, we used the monospecific association between the Hawaiian bobtail squid *Euprymna scolopes* and the beneficial bacterium *Vibrio fischeri* as a model system. For more than a decade, the squid-vibrio system has been used to explore how beneficial symbioses are impacted under simulated and actual microgravity conditions due to its high degree of experimental malleability and rapidly inducible phenotypes (Fig. 1)^23–30^. Briefly, *E. scolopes* have a specialized symbiotic light organ that houses the bioluminescent *V. fischeri* (Fig. 1A). The squid hatch in an aposymbiotic state (i.e., without *V. fischeri*) and must acquire their symbionts horizontally from the surrounding environment^31^. The squid does this with the help of superficial ciliated epithelial appendages (CEA) that circulate the surrounding seawater containing *V. fischeri* towards pores on the surface of the light organ (Fig. 1B)^32^.

During the initiation of the symbiosis the squid’s only immune cell, macrophage-like cells called hemocytes, migrate into the blood sinus of the CEA (Fig. 1C). These structures then undergo bacteria-induced apoptosis (i.e., non-inflammatory cell death) (Fig. 1C) and subsequent regression^33,34^. The normal morphological remodeling of the host light organ tissues is largely driven by the innate immune response to microbial-associated molecular patterns (MAMPs), such as lipopolysaccharide and peptidoglycan (PGN), and is thought to be controlled by the NFκB signaling pathway^35–38^. Under the stress of modeled microgravity conditions, hemocyte migration into the blood sinus of the light organ is delayed compared to gravity controls (Fig. 1E)^26^, suggesting an altered innate immune response, however, the mechanisms underlying this phenotype are unknown.

In this study, the NFκB signaling pathway was explored in the presence and absence of symbiotic microbes as well as under the stress of simulated microgravity conditions. Due to the high cost of spaceflight, many studies exploring the role of microgravity on immune function have relied on simulating low-shear modeled microgravity (LSMMG) conditions using high-aspect-ratio rotating wall vessel bioreactors (HARVs) (Fig. 1D). The HARVs mimic reduced gravity by maintaining the organism(s) in a state of constant fluid suspension under conditions of low fluid shear. The hydrodynamic forces within the HARV offset the effects of gravity within the chamber, such that the squid or bacteria within the HARV chamber “falls” through the liquid medium at a constant terminal velocity. First designed by NASA, this approach has been widely used for more than 30 years to mimic microgravity and has been shown to correlate with results obtained from space flight conditions^39–42^.

Specifically, in this study genes associated with the putative NFκB signaling pathway were identified from the *E. scolopes* genome and transcriptome to assess differential gene expression of the pathway under LSMMG conditions over time in the presence and absence of the symbiont *V. fischeri*. The results of the study revealed those aspects of the pathway that are primarily driven by the onset of the symbiosis and those that were differentially regulated under microgravity-like stress conditions. Together, the results of this study demonstrated how the stress of LSMMG can alter and, in some cases, accelerate the expression of key components of the NFκB signaling pathway, thereby negatively impacting the overall innate immune response of host animals under simulated spaceflight-like conditions.

## Results and discussion

### Elucidation of key NFκB pathway elements in the host *E. scolopes* through data mining

**Figure 2:**
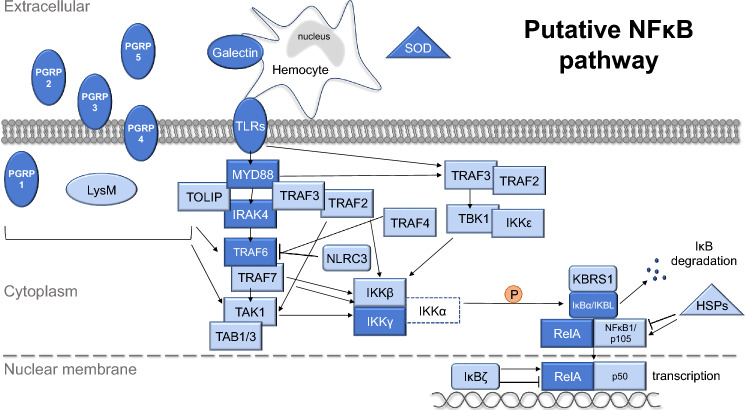
Putative NFκB signaling pathway data mined from *Euprymna scolopes* reference transcriptome and genome. Genes in dark blue have been found previously in *E. scolopes*, whereas genes in light blue are being reported here for the first time. Gene with a dotted line was not found in the *E. scolopes* genome or transcriptome. Elements of the pathway found in *E. scolopes* included pattern recognition receptors (PRRs, oval), effector enzymes (triangle), and signaling cascade genes (rectangular). *PGRP* peptidoglycan recognition receptor proteins *LysM* Lysin motif protein, *TLRs* toll-like receptors *SOD* superoxide dismutase, *HSPs* heat shock proteins, *MYD88* Myeloid differentiation primary response 88, *TOLIP* Toll interacting protein, *IRAK* interleukin 1 receptor-associated kinase, *TRAFs* tumor necrosis factor receptor-associated factors, *NLRC3* NACHT, LRR and CARD domains-containing protein 3, *TAK1* Transforming growth factor beta-activated kinase 1/Mitogen-activated protein kinase kinase kinase 7, *TABs* TGF-beta-activated kinase and MAP3K7-binding protein, *TBK1* Tank-binding kinase 1, *IKKs* Inhibitor of nuclear factor kappa-B kinase, *KBRS1* NFκB inhibitor-interacting Ras-like protein, *IKBL* Inhibitory kappa-B-like, *NLRC3* NLR family CARD domain-containing protein, *IκBs* NFκB inhibitor, *RelA* Transcription factor p65/relish; *NFκB1* Nuclear factor NFκB.

Aspects of the NFκB pathway, including both activators and effectors, have been shown to play important roles in the onset of the squid-vibrio symbiosis^36,43–47^; however, the full pathway has not been previously delineated in *E. scolopes*. To address this issue, innate immune genes associated with NFκB signaling were data mined from the reference transcriptome and genome of *E. scolopes*^48^ to generate a more comprehensive understanding of the NFκB pathway in the bobtail squid (Fig. 2, Table 1). From the reference transcriptome, data mining revealed 74 transcripts corresponding to 47 unique genes that mapped to both canonical and non-canonical signaling paths for NFκB (Fig. 2, Table 1). These recovered genes included pattern recognition receptors (PRRs), effector enzymes, adaptor proteins, and several signaling molecules that work in synergy to initiate transcriptional changes related to the host immune response (Fig. 2, Table 1).

**Table 1:** Targeted genes for NanoString expression assay.

Group	Gene name	SwissProt annotation	Transcript ID	Gene ID
PRR	EsPGRP1^a^	PGRP1_HUMAN2	g32443.t1	cluster_20358
	EsPGRP2	PGRP1_HUMAN	TR392087|c0_g2_i3|m.10497	cluster_4678
	EsPGRP3	PGRP1_BOSIN	c90996_f1p3_2225	cluster_12168
	EsPGRP4^a^	PGRP1_CAMDR	c13944_f1p1_1948	cluster_11708
		PGRP1_CAMDR2	g32454.t1	cluster_11708
	EsPGRP5	PGSC2_DROS	c202596_f11p9_1744	cluster_2407
		PGRP2_HOLDI	g26249.t1	cluster_2407
	EsTLR1	TOLL8_DROME	TR148504|c1_g1_i1|m.29453	cluster_824
		TOLL8_DROME2	c72113_f1p0_2169	cluster_824
	EsTLR2	TLR21_CHICK	TR395607|c0_g1_i2|m.29770	cluster_4721
	EsTLR3	TLR22_CHICK	TR272699|c0_g1_i1|m.41803	cluster_2800
	EsTLR4	TOLL_DROME2	g48290.t1	cluster_22290
	EsTLR5^b^	ALS_MOUSE	g91545.t1	cluster_27922
	EsTLR6^a^	TLR6_MOUSE	g31247.t1	cluster_14365
	EsGalectin1	LEG1_HAECO	TR633522|c3_g2_i1|m.28191	cluster_8370
	EsGalectin2^b^	LEG4_MOUSE	c95712_f1p2_3429	cluster_16338
	LYSM2^a^	LYSM2_DANRE	g11540.t1	cluster_17730
	LYSM3	LYSM3_CHICK	TR298744|c6_g1_i1|m.44844	cluster_3208
Effector enzyme	EsSOD	SODC_BOMMO	TR132802|c5_g2_i1|m.7128	cluster_586
	SODC2^a^	SODC_MOUSE	g77040.t1	cluster_13902
	SODC3^a^	SODC_SCHPO	isotig01040|m.9077	cluster_7124
		SODC_SCHPO2	c124720_f1p94_1276	cluster_7124
	SODM^b^	SODM_RAT	c123132_f1p3_1433	cluster_11217
		SODM_CHAFE	g35029.t1	cluster_20684
	HSP71	HSP7C_RAT	c35333_f1p15_1997	cluster_11716
		HSP7C_RAT2	c36410_f1p4_2104	cluster_11716
		HSP7C_ICTPU	c196330_f1p3_1314	cluster_11716
	HSP90	HS90A_RABIT	g6318.t1	cluster_24185
Signaling	MYD88_1	MYD88_PANTR	c98230_f1p1_1673	cluster_16435
	MYD88_2^b^	MYD88_PANTR2	c141455_f3p1_1351	cluster_530
	EsIRAK4	IRAK4_HUMAN	TR101118|c2_g2_i2|m.40331	cluster_23
	IRAK4_2	IRAK4_BOVIN	c8160_f6p3_2517	cluster_15833
		PBL6_ARATH	g88227.t1	cluster_15833
	TRAF2	TRAF2_HUMAN	g53219.t1	cluster_5541
	TRAF3	TRAF3_MOUSE	c82312_f1p0_2491	cluster_15867
	TRAF4	TRAF4_MOUSE	TR683727|c9_g1_i5|m.7586	cluster_9167
		TRAF4_MOUSE2	c43236_f1p0_1788	cluster_9167
	EsTRAF6	TRAF6_BOVIN1	g38415.t1	cluster_4580
		TRAF6_BOVIN2	TR385091|c1_g1_i2|m.21623	cluster_4580
		M3K7_PONAB	c55278_f2p1_2609	cluster_16222

^a^ Probes were unsuccessful in the NanoString assay.

^b^ Not targeted for NanoString analysis.

As bacteria colonize the host light organ they release MAMPs that are recognized by host PRRs and trigger several innate immune-related developmental changes in the host symbiotic light organ (Fig. 1E)^43^. Several of the PRRs identified in this study have been previously observed in *E. scolopes*, such as peptidoglycan recognition proteins (EsPGRPs), galectins (EsGalectin1 and 2), and Toll-like receptors (EsTLR1-6)^36,45,46,49–51^ (Fig. 2, Table 1). PGRPs are highly conserved PRRs that recognize the PGN component of bacterial cell walls^52^ and previous studies on the *E. scolopes* PGRPs indicate that they are involved in NFκB signaling within the host light organ^36,50^. Examination of the reference transcriptome identified all five of the previously reported PGRPs (EsPGRP1-5)^36,45^. No additional PGRPs were found. Analysis of the PGRP sequences showed that EsPGRP1-3 and EsPGRP5 contained signal peptide sequences, whereas EsPGRP4 harbored two transmembrane helix domains suggesting it may be membrane-bound rather than secreted (Fig. 2; Dataset S1). All the EsPGRPs had the characteristic amidase activity domain and the necessary zinc-binding residues involved in the breakdown of PGN (Dataset S1). Understanding the type of PGN that PGRPs bind to can provide insight into how the squid may detect and respond to different bacterial MAMPs. Additional examination of the EsPGRP catalytic domains suggested that EsPGRP1, 2, and 3 may bind specifically to DAP-type PGN, the dominant form of PGN for gram-negative bacteria, whereas the specific binding capacity of EsPGRP4 and 5 was indeterminable based on the recovered sequences (Fig. S1).

Other PRR categories that were screened in the reference transcriptome and genome included galectins, Toll-like receptors (TLRs), and LysM binding proteins. Galectins are carbohydrate recognition proteins known to bind to microbial surfaces for bacterial recognition and subsequent phagocytosis^45,47,53–56^. Only two galectins were recovered from the reference transcriptome and genome, both of which have been previously reported in the *E. scolopes* macrophage-like innate immune cells, hemocytes (Fig. 2), yet their involvement in the NFκB pathway is not well understood^49^. Additionally, six TLRs (EsTLR1-6) were observed, all of which have been previously reported in *E. scolopes*^46^. TLRs are hypothesized to initiate the NFκB pathway by recognizing MAMPs and subsequently interacting with the Toll/interleukin-1 receptor (TIR) domain of signaling genes, such as MYD88 (Fig. 2, Table 1). Additionally, two previously unreported LysM proteins (LysM2 and 3) were observed in the *E. scolopes* reference transcriptome and genome (Fig. 2, Table 1). These PRRs were characterized by repeating lysin motifs that have been shown to recognize PGN as well as chitin in plants, fungi, and bacteria^57–61^. Analysis of the LysM sequences did not reveal a signal peptide or transmembrane helix domains suggesting the proteins are confined to the cytoplasm (Fig. 2).

In addition to PRR activators of the NFκB pathway, the analysis revealed several effector enzymes that can modulate the NFκB pathway, including previously unreported isoforms of superoxide dismutase (SOD) and heat shock proteins (HSPs) (Table 1). In hatchling *E. scolopes*, the crypt spaces within the light organ become oxidatively stressful preventing non-symbiotic bacteria from entering. The squid then utilizes SOD to combat the release of ROS^49^. Three SODs were recovered that contained copper/zinc-binding domains, including previously identified EsSOD, while a fourth SOD was found with a manganese/iron-binding domain (Table 1, Dataset S1). Additionally, two heat shock proteins, HSP71 and HSP90, were also recovered from the *E. scolopes* genome and transcriptome that have been shown to modulate aspects of NFκB signaling in other cephalopods^62^.

Stimulation of PRRs and effector enzymes activates the recruitment of adapter proteins and protein kinases, such as MYD88 and IRAK, respectively^63^. Although both genes have been previously reported in *E. scolopes*^36^, several new isoforms were recovered from the reference transcriptome and genome (Table 1). Once activated, IRAK can then dissociate from MYD88 and interact with ubiquitin ligases, such as TNF receptor-associated factors (TRAFs), which have been shown to polyubiquitinate themselves and other proteins (e.g., IKKγ). These ubiquitinated proteins then recruit protein kinase complexes (e.g. TAK1 and TABs)^64^ that then activate the IκB kinase (IKK) complex for NFκB driven transcription. Genes associated with the TAK1/TAB and IKK complexes were recovered in *E. scolopes* (Fig. 2, Table 1). The IKK complex generally consists of three proteins: two catalytic components, IKKβ and IKKα, and a regulatory component, IKKγ, the latter of which was previously reported in *E. scolopes*^36^. Interestingly, our analysis revealed transcripts and genes associated with IKKβ but not IKKα. The absence of IKKα in the *E. scolopes* genome and reference transcriptome was surprising as it has been observed in other cephalopods, such as *Octopus vulgaris* and *Haliotis discus hannai*^65,66^. These results suggest that the IKK complex in the *E. scolopes* may exhibit an alternate configuration and may not follow the canonical pathway NFκB activation^67^.

Next, the activated IKK complex typically phosphorylates the NFκB inhibitor, IκBα, which is then polyubiquitinated and targeted for proteasomal degradation. Once the NFκB subunits are freed from IκB they can translocate into the nucleus and initiate transcription. Our analysis recovered sequences associated with the most common forms of the transcription factors in *E. scolope*s including RelA and NFκB1/p50 (Fig. 2, Table 1). Other putative inhibitor proteins were also recovered from *E. scolopes* in this analysis including IKBL, KBRS1, NLRC3, and the IκBα related gene IκBζ, all of which have been shown to regulate NFκB transcription by affecting other components in the signaling pathway (Fig. 2, Table 1)^68–70^. Additional NFκB associated signaling transcripts that were found in this analysis of the *E. scolopes* transcriptome and genome included IKKε, TBK1, TOLIP, additional TRAFs (TRAF2, 3, 4, and 7), TAK1, and TAB1/3 (Fig. 2, Table 1) suggesting that, except for IKKα, *E. scolopes* harbors all the canonical elements of the NFκB pathway.

### Symbiosis state was the major driver of differential gene expression in the host NFκB pathway

To begin to explore how simulated microgravity alters the expression of the NFκB pathway in the *E. scolopes* light organ, hatchling animals were incubated in the HARVs in both the LSMMG and gravity control positions in the presence and absence of the symbiotic *V. fischeri* for 2, 6, 8, 10, 12, 16, 18, 24 h. Total RNA extracted from host light organs was then screened for differential expression of 33 genes associated with the NFκB pathway using NanoString nCounter direct transcript counts (Table 1; Dataset S2)^71^. Note due to space limitation in the NanoString 96-well plate configuration there was no symbiotic control for the 24 h LSMMG treatment. Genes were considered significantly differentially expressed (DEGs) at an adjusted p-value cutoff of 0.10, and highly significant at a cutoff of 0.05 (Dataset S3).

**Figure 3:**
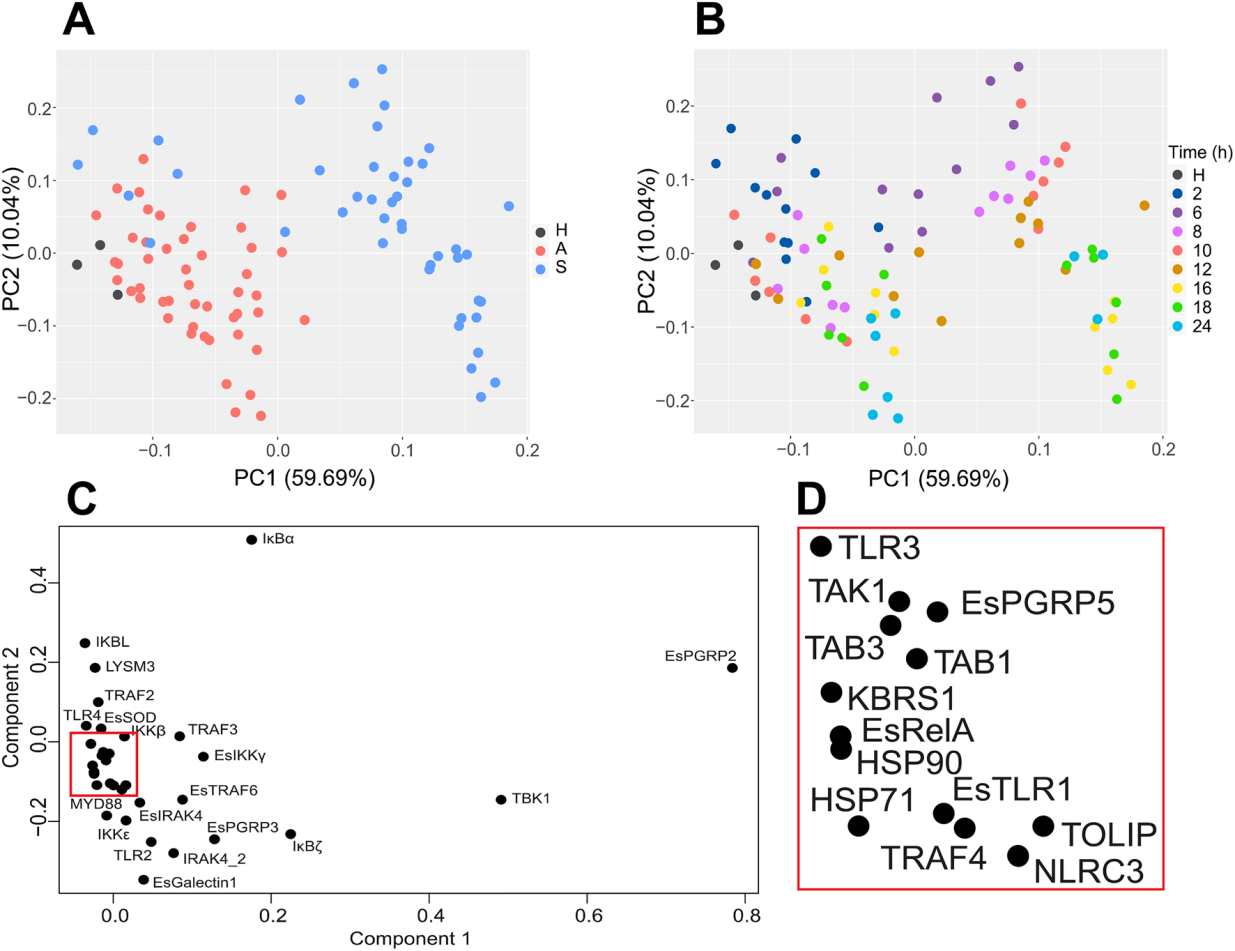
Principal component analysis (PCA) of all replicate samples exposed to both simulated microgravity and gravity controls. (**A**) Samples organized by symbiosis state including newly hatched animals (black), aposymbiotic animals (red) maintained without *V. fischeri* and symbiotic animals (blue) colonized with *V. fischeri*. (**B**) Corresponding PCA color-coded by time in hours (h) depicting a progression of differential gene expression over time. (**C**) Loading plots of the PCA depicting the specific genes driving the differential expression within the host animal. A cluster of genes within the loading plot is expanded in (**D**).

**Figure 4:**
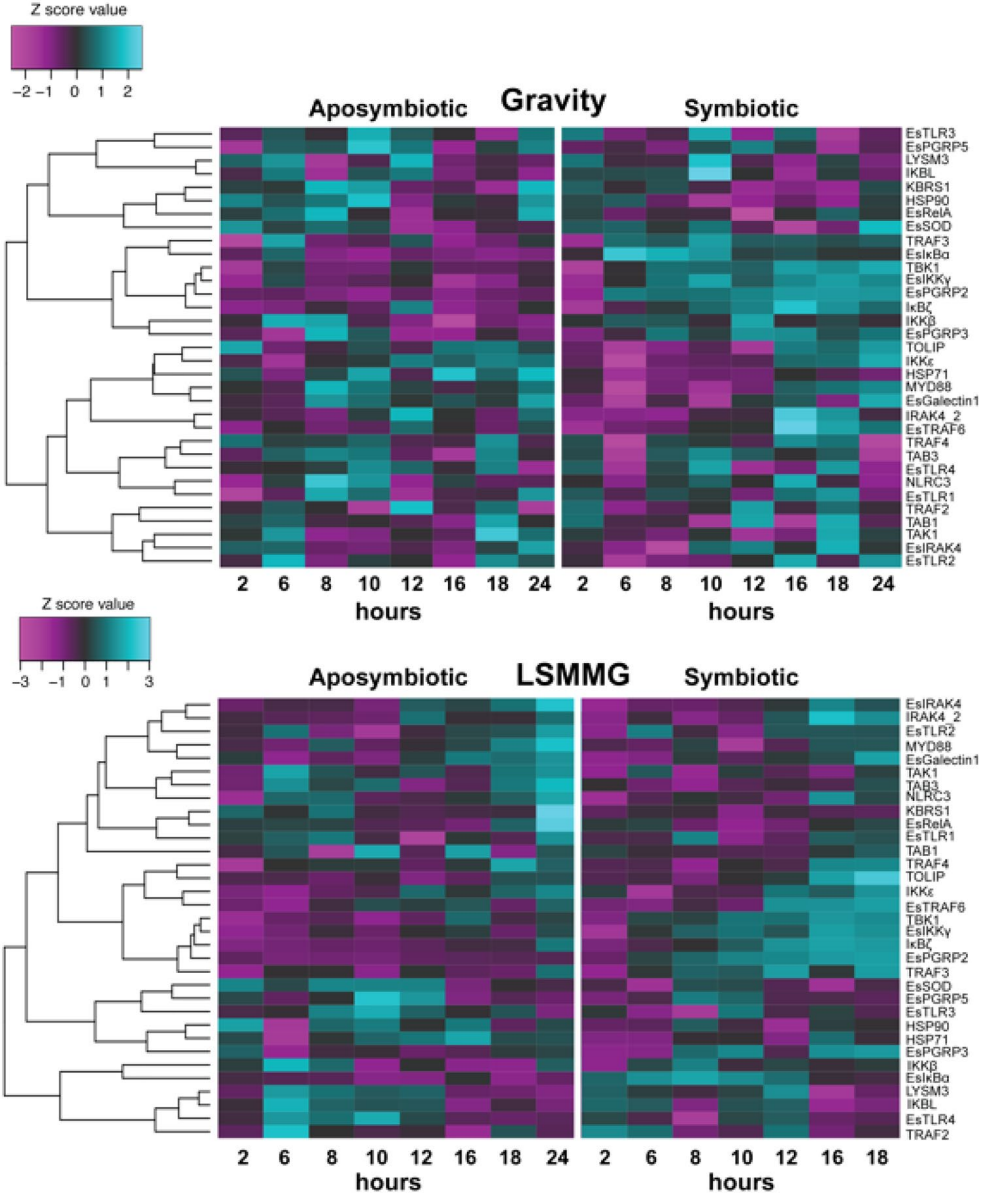
Heatmap of voom transformed (log-CPM) NanoString gene expression hierarchically clustered by dissimilarities across time and by gravity or low-shear modeled microgravity (LSMMG) treatment. The teal color indicates upregulated expression whereas purple color represents downregulated expression in that associated condition. A detailed description of the targeted genes is listed in Table 1.

Analysis of animals exposed to both LSMMG and gravity controls revealed that the symbiosis state was the major driver of differential gene expression of the NFκB pathway rather than the stress of simulated microgravity (Figs. 3, 4). Principal component analysis (PCA) revealed that 59.69% of the variation observed in the expression of NFκB pathway genes was due to the onset of the symbiosis (Fig. 3A). A similar trend is also visualized by heatmap analysis (Fig. 4, Figs. S2–S4). The overall shift in differential expression in symbiotic animals occurred 6 h post-inoculation as those animals exposed for only 2 h to *V. fischeri* clustered with the hatchling and aposymbiotic animals (Fig. 3B).

Of the different genes examined, PCA loading plots revealed that the differential expression of the PRR EsPGRP2 and signaling gene TBK1 had the largest influence on the onset of the symbiosis (Fig. 3C). EsPGRP2 is one of the most well-studied PGRPs in *E. scolopes* and has been shown to localize to the cytoplasm of the CEA where it is later secreted into the extracellular mucus and within the crypt spaces of the light organ in response to *V. fischeri* colonization^51^. EsPGRP2 has also been shown to be expressed in the accessory nidamental gland, the other symbiotic organ in female *E. scolopes* used to secrete anti-fouling bacteria during squid egg production^72,73^. EsPGRP2 is thought to help control potentially harmful excess PGN produced by *V. fischeri* or other bacteria by breaking it down using its amidase catalytic domain^51^. Except for 2 h, EsPGRP2 was significantly upregulated in symbiotic animals at all time points compared to aposymbiotic controls in both modeled microgravity and gravity conditions (Fig. 4, Figs. S2–S4, Dataset S3). These results support EsPGRP2 importance during the early colonization stages of *V. fischeri* (Dataset S1).

The other most influential symbiosis-associated gene targeted in this study was TANK Binding Kinase 1 (TBK1), a noncanonical kinase in the IKK family that plays an important role in regulating animal innate immune responses, apoptosis, tumorigenesis, and development^74–77^. Although its role in the squid-vibrio symbiosis is not yet clear, TBK1 was significantly upregulated in all symbiotic animals beginning 6–8 h post-inoculation (Fig. 4, Figs. S2–S4, Dataset S3). Recent research in mammalian systems has indicated that TBK1, along with its analog IKKε, can sense bacterial infections through multiple signaling pathways and may be activated through upstream MAMP-driven IKK phosphorylation, as well as by trans-autophosphorylation^77,78^. In mammalian systems, TBK1 can also be directly activated through TLR2 and MYD88 activation to mobilize transcription factors associated with the NFκB pathway^77,79^.

**Figure 5:**
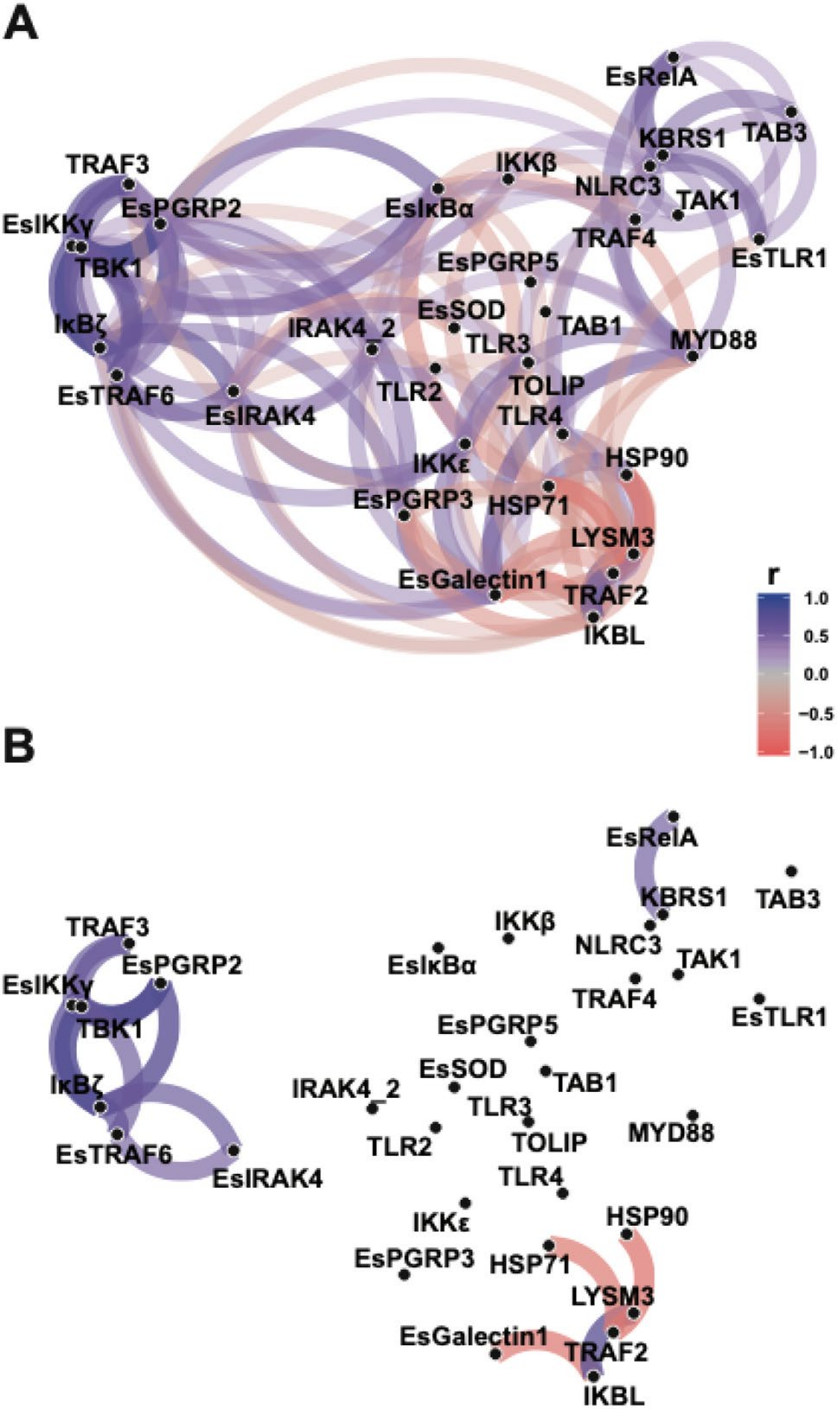
Network plot of Pearson correlation of voom transformed (log-CPM) NanoString assay gene expression counts to visualize gene expression relationships. Genes that are more highly correlated are closer together according to multidimensional clustering of the absolute values of correlation and are joined by stronger paths. Blue paths indicate positively correlated genes whereas red indicates negatively correlated genes. (**A**) all correlations with r-value ≥ 0.40. (**B**) highest correlations with minimum correlation r-value ≥ 0.6.

In the *E. scolopes* light organ, analysis of the gene expression patterns indicated that TBK1 expression was highly correlated with PRR EsPGRP2, adaptor protein TRAF3, and regulatory kinases EsIKKγ and IκBζ (Fig. 5, Dataset S4). Similar to the kinase TBK1, EsIKKγ plays an important role in NFκB signaling and assembly kinase complexes^80^. EsIKKγ contains the NFκB essential modulator (NEMO) domain with polyubiquitination binding sites and the C2H2 (classical)-type/ ‘NEMO’ type zinc finger thought to interact with the other IKK subunits (Dataset S1). EsIKKγ was also significantly upregulated in symbiotic light organs at 10, 16, and 18 h regardless of gravity state suggesting that, together with TBK1, these regulatory kinases may be playing an important role in symbiosis-activation of the host NFκB pathway (Dataset S3).

In addition to the regulatory kinases, TRAF3 expression was also strongly correlated to TBK1 and EsIKKγ (Fig. 5). TRAFs play an important role in innate immunity by interacting with adapter proteins or other kinases to continue signaling cascades by polyubiquitination, which can then recruit other important kinases, such as TBK1, TAK1, and IKKs^77^. TRAF3 is one of the most functionally diverse of the TRAFs in particular in the regulation of non-canonical NFκB signaling pathways^81^. This is the first report of TRAF3 in *E. scolopes* and the sequences not only contained the usual TRAF domains but also had a seven in absentia (SIAH)-type zinc finger that is important in E3 ubiquitin ligases (Dataset S1). The SIAH-type zinc finger is a mammalian homolog to the SINA-type zinc finger first found in *Drosophila* that can activate NFκB pathways^82^. In symbiotic animals TRAF3 gene expression was variable between 2 and 8 h post-inoculation, however, the expression became consistently upregulated in symbiotic animals starting at 10 h (Dataset S3). TRAF3 function is not well understood in invertebrates, however, several studies have shown that TRAF3 can respond to viral and bacterial challenges^83,84^. The strong correlation of TRAF3 expression with TBK1 and IKKε (Fig. 5) suggests it may mediate the activation of a complex between TBK1 and IKKε, although additional research is needed for confirmation.

### Impact of low shear modeled microgravity on NFκB pathway gene expression

**Figure 6:**
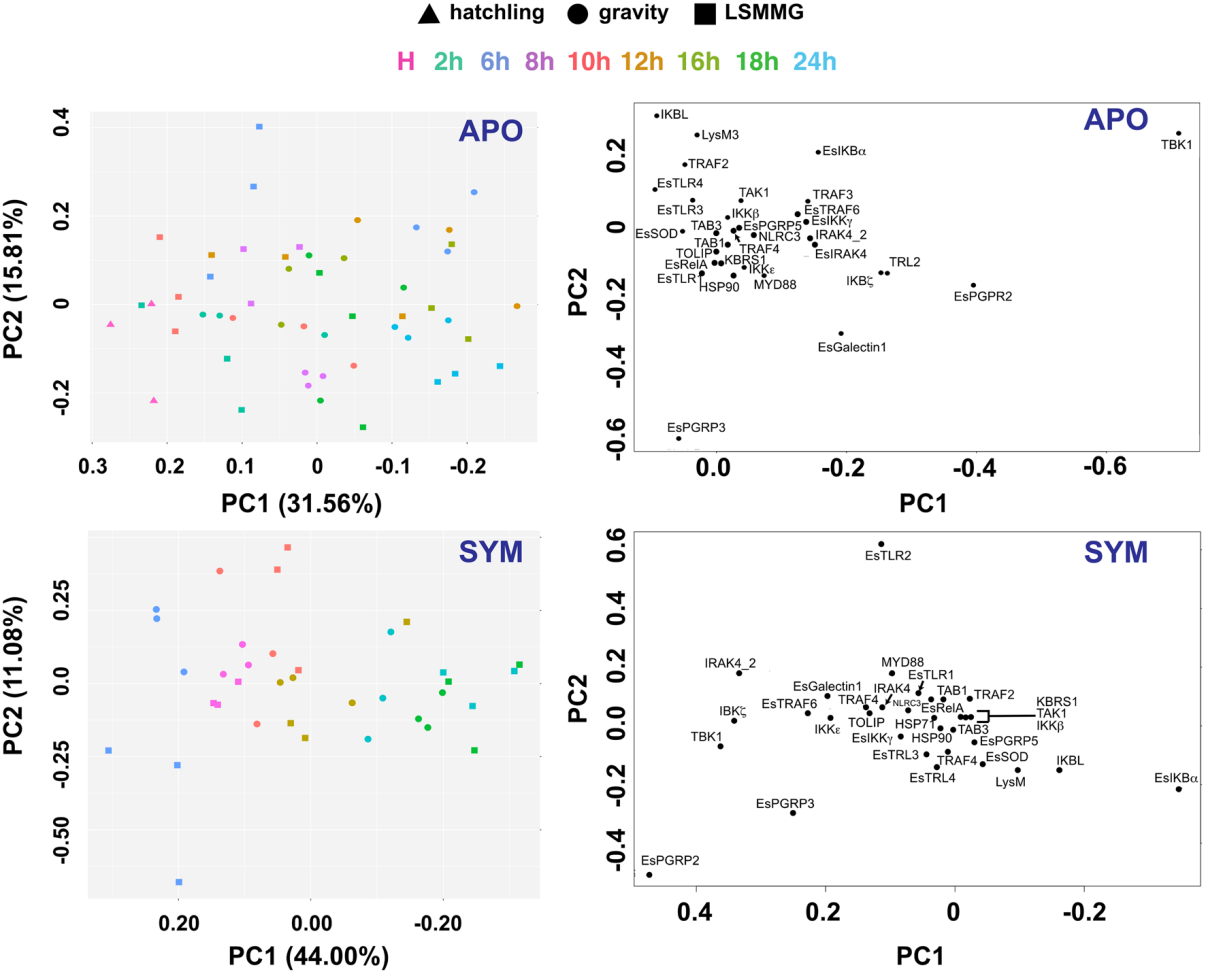
Principal component analysis (PCA) of NanoString gene expression assay samples separated by symbiosis state and HARV treatment. Aposymbiotic (APO) plots include the untreated hatchling animals (triangle) as well as the gravity (circle) and LSMMG (square) treatments. To improve visualization of the data over the developmental timeline, 2 h symbiotic (SYM) data were not included in the PCA and loading plots.

To explore these results further, samples were separated based on symbiosis state to explore the impact of bacterial colonization and modeled microgravity stress over time on NFκB pathway gene expression in the host squid (Fig. 6, Figs. S5 and S6). Analysis of the NanoString expression data suggested that several genes associated with the NFκB pathway were differentially expressed under the LSMMG treatments compared to gravity controls and that some gene expression was occurring earlier in the targeted developmental timeline in both aposymbiotic and symbiotic conditions (Table 2, Fig. 4, Dataset S3, Figs. S2–S4). For example, under normal gravity conditions, differential expression of NFκB pathway genes did not occur until 6 h post inoculation (Dataset S3; Figs. S2–S4), whereas under LSMMG conditions several genes including LysM3, TRAF2, TRAF4, IKKε, and IκBα, and IKBL were all significantly upregulated in symbiotic animals beginning 2 h post inoculation (Table 2, Dataset S3). Additionally, other genes including TBK1, IκBζ, EsPGRP3, and EsTRAF6 were also up-regulated earlier compared to the gravity controls (Table 2, Dataset S3). These results suggest that the stress of modeled microgravity may be triggering elements of the NFκB signaling pathway earlier than under gravity controls.

**Table 2:** Temporal changes of significantly up-regulated genes in aposymbiotic animals relative to symbiotic animals under gravity and low-sheared modeled microgravity (LSMMG) conditions with adjusted p-value ≤ 0.10^a^.

	2h		6 h		8 h		10 h		12 h		16 h		18 h	
Treatment	APO	SYM	APO	SYM	APO	SYM	APO	SYM	APO	SYM	APO	SYM	APO	SYM
Gravity	n/a	n/a	TRAF4	EsPGRP2	MYD88	EsPGRP2	MYD88	EsPGRP2	IRAK4	EsPGRP2	HSP71	EsPGRP2	n/a^b^	EsPGRP2
			MYD88	IκBα	HSP90	TBK1	HSP90	TBK1	LysM3	TBK1		TBK1		TBK1
						IκBα	HSP71	IκBα	IKKε	IκBα		IκBζ		IκBζ
						EsIKKγ	EsPGRP5	IκBζ		IKKβ		IκBα		IκBα
						IKBL	EsGalectin1	EsIKKγ				EsPGRP3		IRAK4
								TRAF3				EsTRAF6		EsIKKγ
								LysM3				EsIKKγ		EsTRAF6
								IKBL				TRAF3		
								TRAF2						
LSMMG	EsPGRP3	*IκBα*	IKBL	EsPGRP2	IKBL	EsPGRP2	EsTRAF6	EsPGRP2	EsPGRP5	EsPGRP2	n/a	EsPGRP2	n/a	EsPGRP2
	*EsGalectin1*	*TRAF2*	TRAF2	*TBK1*	TLR3	IκBα	HSP90	TBK1	HSP90	TBK1		IκBζ		TBK1
	*HSP90*^*c*^	*LysM3*	LysM3	IκBα	TLR4	TBK1	**MYD88**	IκBα	HSP71	IκBα		EsPGRP3		IκBζ
	*IRAK4*	*IKBL*	TAB3	HSP90	TAK1	*IκBζ*		*EsPGRP3*	EsSOD	IκBζ		IRAK4		EsPGRP3
	*HSP71*	TRAF4	EsSOD		**TRAF4**			IκBζ		*EsTRAF6*		TBK1		IκBα
	EsSOD	**IKKε**						**EsIKKγ**		**TRAF3**		EsIKKγ		EsTRAF6
								TRAF3		TRAF2		TOLIP		IRAK4
														EsIKKγ
														TRAF3
														TOLIP

^a^Fold changes and adjusted p-values listed in Dataset S2.

^b^Indicates no significantly differentially expressed genes at this time point.

^c^Genes in italics exhibit earlier temporal expression under LSMMG, whereas expression of genes in bold were delayed under LSMMG conditions relative to gravity controls.

Interestingly, two of the differentially expressed genes, IKBL and IκBα, are homologs that both contain ankyrin repeats and serve as inhibitors of NF-κB signaling by blocking nuclear localization and transcriptional activity of RelA (i.e., p65)^85^. The increased expression of the IKBL and IκBα inhibitors earlier in the bacteria-induced developmental timeline under LSMMG conditions may be associated with the observed delay in innate immune cell activation and trafficking^26^. Normally, under gravity conditions, MAMP shedding typically occurs through the release of outer membrane vesicles (OMVs) by *V. fischeri*^86^, and the host responds by trafficking macrophage-like hemocytes into the blood sinus of the CEA within 1–2 h after exposure to *V. fischeri*. In simulated microgravity conditions, however, there is a delay of up to 8–10 h in the activation of hemocyte trafficking into the CEA blood sinus^26^, even though there is an LSMMG-induced increase in OMV and MAMP release by *V. fischeri*^29^. This disparity in the host response to the elevated MAMPs under simulated microgravity conditions may be correlated to the increase in expression of the IKBL and IκBα inhibitors, thereby delaying NF-κB signaling and downstream gene transcription (e.g., activation of cytokines). Additionally, IKBL gene expression was positively correlated to TRAF2 and LysM3 expression and negatively correlated to EsGalectin1 expression (Fig. 5, Dataset S4). This correlation coupled with the up-regulation of IKBL, TRAF2, and LysM3 expressed in aposymbiotic animals at 6 h (Table 2; Dataset S3) suggests these components of the NF-κB pathway may be stress-inducible, thereby contributing to the delay in innate immune system activation under modeled microgravity stress.

Although there have been no reports of differential expression of LysM under natural or simulated microgravity conditions, differential expression of TRAF2 has been observed using alternative modeled microgravity platforms including Random Positioning Machines and hind-limb unloading^87,88^. In symbiotic animals, TRAF2 was significantly expressed (*p* ≤ 0.5) under LSMMG conditions at 2 h (Table 2, Dataset S3). TRAF2 is important in several signaling cascades, including NFκB, oxidative stress-induced apoptotic cell death, and DNA damage responses^89–92^. TRAF2 can both positively and negatively regulate stress-induced cell cytotoxicity and is highly context-dependent^92^. Additional research will be required to explore the elevated TRAF2, LysM, and IKBL levels and ascertain whether there are specific interactions between these molecules under LSMMG-induced stress.

Additionally, in the aposymbiotic animals, there was a significant increase in the expression of the effectors HSP90, HSP71, and EsSOD, as well as the PRR EsGalactin1 under LSMMG compared to the symbiotic animals. Although the HSPs and SODs are involved in the NFκB pathway, these genes are likely being upregulated as a novel stress response to the simulated microgravity conditions as these genes were not differentially expressed under gravity controls. Numerous studies have shown that HSPs, including HSP90 and HSP70, and SODs are upregulated in both plants and animals in response to spaceflight conditions including microgravity and radiation^93–98^. Additionally, the increased expression of EsGalectin1 in the aposymbiotic animals may also reflect a stress response. Although the increase of galectin-1 expression has not been previously shown to be increased under spaceflight or simulated microgravity conditions, it has been shown to prevent oxidative stress responses in a wide range of animal hosts and may reflect a more broad stress response in the host squid^99,100^. Interestingly, the increase in these stress-response genes was only observed in the aposymbiotic animals and may suggest that the host may be sensing the *V. fischeri* in symbiotic animals and overriding some of the LSMMG-induced stress responses in the host tissues.

Not all targeted genes exhibited earlier differential expression, in fact, several genes, such as TRAF3, TRAF4, MYD88, and EsIKKγ, exhibited a delay in expression over time under LSMMG conditions. The delayed expression of these genes was not correlated with each other (Fig. 5) but may signify a disruption to the normal signaling pathway under LSMMG conditions that contributes to the overall delay in the host innate immune response (Fig. 1E). For example, TRAF3 is a key regulator of TLRs^101^. Additionally, TRAF3 can form a complex with protein kinases TBK-1 and IKKε that leads to the activation of type I interferon^102,103^. The delayed expression of TRAF3 may impede the complex formation thus dysregulating the host immune response. Additional experimentation will be required to determine the precise function of TRAF3 in *E. scolopes* under both LSMMG and normal unit gravity conditions.

In summary, the results of this study provided a more comprehensive understanding of the NFκB signaling pathway in *E. scolopes* during the onset of symbiosis and showed that many genes of the pathway were differentially expressed under the stress of modeled microgravity conditions. Although similar dysregulation has been observed in a wide range of host animals under simulated and natural spaceflight conditions^3,104,105^, this study provides new insight into how the onset of a beneficial symbiosis under these LSMMG conditions can impact the host innate immune transcriptional responses. The results showed that the major driver of differential gene regulation of the NFκB pathway was the onset of the symbiosis, suggesting that interactions with microbes may trigger key developmental and innate immune responses that, in some cases, supersede microgravity-induced responses. However, some key elements associated with inhibiting the NFκB pathway were upregulated within a few hours of exposure to simulated microgravity conditions indicating that the stress of microgravity can impose rapid physiological changes within the host animal in the presence and absence of beneficial microbes. Additionally, the multi-functional role of many elements of the NFκB signaling pathway in other key pathways, such as apoptosis and crosstalk with the microbiome, likely contributed to the dysregulation observed in the pathway, confounding the elucidation of host innate immune responses during the stress of simulated microgravity. These results do, however, expose the importance of examining host-microbe interactions in situ and in real-time to more fully understand the innate immune response of animals during spaceflight conditions.

## Materials and methods

### Data mining of the host reference transcriptome and genome

Gene sequences relating to the putative innate immune NFκB pathway in *E. scolopes* were found by data mining the reference transcriptome and fully assembled genome^48^. Translated genes were subsequently searched in the NCBI non-redundant databases using the Basic Local Alignment Search Tool for proteins (BLASTp) to confirm their annotation. The gene level homology was examined as expected values (i.e., E-values) and all hits had E-values < − 45. Domain architecture for each gene was confirmed through InterProScan^106^ (Dataset S1). Genes that have been found previously in *E. scolopes* were given the ‘Es’ notion in this study to maintain consistency with previously published literature.

### Animal husbandry and general procedures

All cephalopod procedures were approved by both the University of Florida (approval number 201910899) and Kennedy Space Center (approval number GDR-20-128) Institutional Animal Care and Use Committees and were performed in accordance with the approved protocols and guidelines. Mature *E. scolopes* squid were maintained in aquaria within an environmental growth chamber at 23 °C on a 12 h light/dark cycle. Clutches of eggs were removed from the adult tanks and incubated separately in individual aquaria for their full developmental cycle (~ 21 days). After hatching, the juvenile squid were maintained in filtered-sterilized seawater (FSW) as either aposymbiotic (i.e., no symbiosis-competent *V. fischeri*) or rendered symbiotic. For symbiotic treatments, animals were inoculated with 1 × 10^5^ cells of *V. fischeri* ES114 per ml of FSW. The concentration of *V. fischeri* was determined spectrophotometrically (A_600nm_) as an OD of 1 corresponds to 4 × 10^8^ cells per ml of culture, as previously determined by plate counts^107^. In all treatments, the onset of symbiosis was monitored by luminescence levels using an ATP photometer (GloMax 20/20 Luminometer, Promega, Corps., Madison, WI).

### Simulated low-shear modeled microgravity (LSMMG) conditions

To simulate the LSMMG conditions, a rotary culture system was used with 16 replicate 50-mL volume high-aspect ratio vessels (HARVs; Synthecon, Houston, TX) and rotated at a constant 13 rpm (Fig. 1D). The HARVs were either rotated around a horizontal axis to simulate LSMMG or a vertical axis to serve as a normal gravity (1 × g) control as previously described^25,40^. For all time points, gravity controls were conducted in HARVs where the axis of rotation was perpendicular to the test system to ensure that the phenotypes detected were not simply the results of being in the HARV system. Both LSMMG treatments and gravity controls were run simultaneously within a environmental growth chamber on a 12 h light/dark cycle and maintained at a constant 23 °C.

Juvenile squid were kept aposymbiotic or symbiotic as described above. The animals were added to the HARVs through an opening on the surface of the HARVs and then sealed with zero headspace. A semipermeable membrane provided aeration for the host and symbiont. Previous studies have shown no change in oxygen levels during animal and bacteria co-incubations^30^. A total of four replicate animals were incubated in each HARV vessel for 2, 6, 8, 10, 12, 16, 18, or 24 h and then animals were removed and immediately flash frozen in liquid nitrogen and stored at − 80 °C. Additionally, a subset of animals were collected within 5 min (i.e., 0 h) of hatching and frozen in liquid nitrogen for comparison purposes.

### NanoString target gene probe design

Target genes were chosen from the data mining results to give an overall representation of elements of the NFκB pathway in *E. scolopes*. Optimal fluorescently tagged probes of 100 bp length were designed and synthesized at NanoString Technologies, Inc. in Seattle, WA for the selected genes to ensure no off-target binding. Probes were designed to hit whole genes and not specific isoforms. There were a total of 33 target genes and two housekeeping genes designed and successfully assayed by NanoString gene expression technology (Table 1; Table S1).

### RNA extraction and gene expression analysis

Total RNA was extracted in triplicate from dissected light organs using RNeasy and Qiashredder kits according to the manufacturer’s protocol (Qiagen, Germantown, MD). A minimum of three light organs was used for each replicate RNA extraction. RNA was pooled from a minimum of three separate extractions derived from three different technical replicates of HARV exposure to increase overall genetic variability and to ensure that gene expression results were not based on variability between egg clutches.

RNA quantity was assessed using Qubit 2.0 (Thermo Fisher Scientific, Waltham, MA) and RNA quality was assessed with a 2100 Bioanalyzer (Agilent Technologies, Santa Clara, CA). All samples were normalized to 20 ng per µl of RNA and 100 ng of RNA was run for each sample on the NanoString nCounter MAX system (NanoString Technologies, Seattle, WA). Note due to space limitations in the NanoString 96-well sequencing plate, 24 h LSMMG-treated symbiotic animals were not able to be sequenced and were thus not included in some of the data analysis.

Data were filtered and normalized using nSolver analysis software (v4.0; NanoString Technologies). Background subtraction was performed using the geometric mean of the negative controls and data was normalized using the geometric mean of positive controls for every time point and housekeeping genes (*actB3* and *pyc1*). Voom transformation from the limma package (v3.8) in R was applied to the normalized counts. This transformation used the empirical Bayes method by pooling estimates of sample variance to assess the expression level variance within samples and transform data into log2-counts per million (CPM). This method has been shown to be more useful for smaller sample size studies, such as with NanoString assays^108,109^. The voom transformed data were passed to a linear model in the limma package to remove the mean–variance relationship and assess statistically significant differentially expressed genes^109^. Significance values were adjusted according to the Benjamini–Hochberg multiple test correction procedure and considered significantly differentially expressed at adjusted p-values < 0.10 and highly significant at < 0.05. Several R packages were used to further analyze and visualize the expression results including heatmap.2 from gplots, Pearson network correlation analysis using the package ‘corrr’ from ggplot2 and ggraph, and the prcomp() function was used for principal component analysis (PCA). All raw NanoString data generated in this study is available at the National Center for Biotechnology Information (NCBI) Gene Expression Omnibus (GEO) under accession number GSE247559.

### ARRIVE statement

All animal experiments were performed in accordance with ARRIVE guidelines (Animal Research: Reporting of In Vivo Experiments). For example, all cephalopod procedures were approved by both the University of Florida (approval number 201910899) and Kennedy Space Center (approval number GDR-20-128) Institutional Animal Care and Use Committees and were performed in accordance with the approved protocols and guidelines.

### Supplementary Information

Supplementary Information 1.

Supplementary Information 2.

Supplementary Information 3.

Supplementary Information 4.

Supplementary Information 5.

## Data Availability

All data generated as part of this study is available within this manuscript, including figures, and supplemental materials. Additionally, the NanoString data generated in this study has been submitted to the National Center for Biotechnology Information (NCBI) Gene Expression Omnibus (GEO) under accession number GSE247559.
