# Supplementary material for: Impact of modeled microgravity stress on innate immunity in a beneficial animal-microbe symbiosis
Source: Sci Rep. 2024 Feb 5;14:2912. doi: 10.1038/s41598-024-53477-3 (PMC10844198; doi:10.1038/s41598-024-53477-3)

Dataset S4. Matrix of Pearson correlation of voom transformed (log-CPM) NanoString assay gene expression counts to visualize gene expression relationships between different genes. Histograms show the correlation of each gene expression for all samples. Number size corresponds to correlation level. pvals = "***" ≤ 0.001, "**" ≤ 0.01, "*" ≤ 0.05, "." ≤ 0.1, & " " ≤ 1.

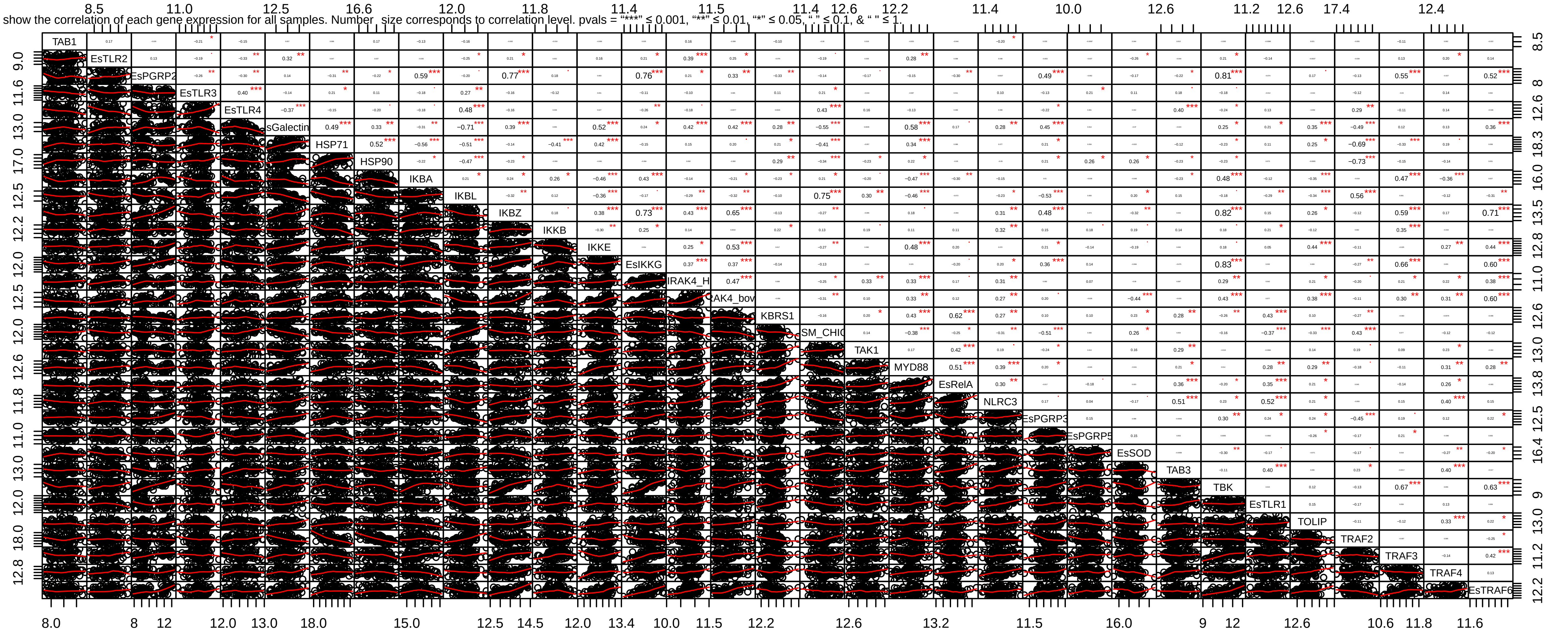

Supplement: Supplementary file 4 — Supplementary Information 4. [file 41598_2024_53477_MOESM4_ESM.pdf]
