# Supplementary material for: Impact of modeled microgravity stress on innate immunity in a beneficial animal-microbe symbiosis
Source: Sci Rep. 2024 Feb 5;14:2912. doi: 10.1038/s41598-024-53477-3 (PMC10844198; doi:10.1038/s41598-024-53477-3)
Supplement: Supplementary file 5 — Supplementary Information 5. [file 41598_2024_53477_MOESM5_ESM.pdf]

Supplemental Materials

Impact of modeled microgravity stress on innate immunity in a beneficial animal-microbe symbiosis

Alexandrea A. Duscher, Madeline M. Vroom, and Jamie S. Foster*

Department of Microbiology and Cell Science, Space Life Science Lab, University of Florida, Merritt Island, FL 32953, USA

*Corresponding author: jfoster@ufl.edu
ORCID: 0000-0001-8603-4006

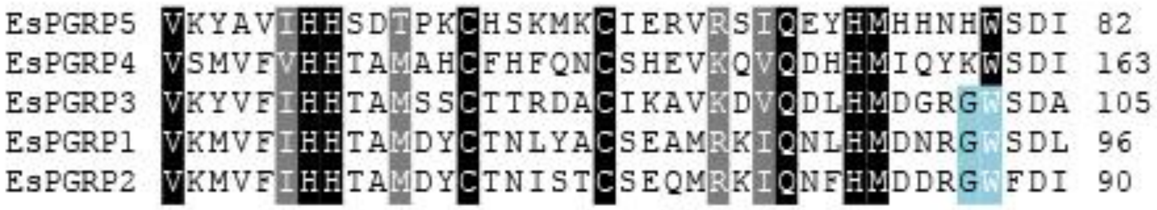

Figure S1. Alignment of translated *E. scolopes* peptidoglycan recognition receptor proteins (EsPGRP) transcripts to identify specific catalytic residues. Black highlighted amino acids indicate conserved amino acid residues. Grey highlight amino acids indicate slightly conserved amino acid residues. Blue highlighted amino acids indicate putative binding to DAP-type peptidoglycan.

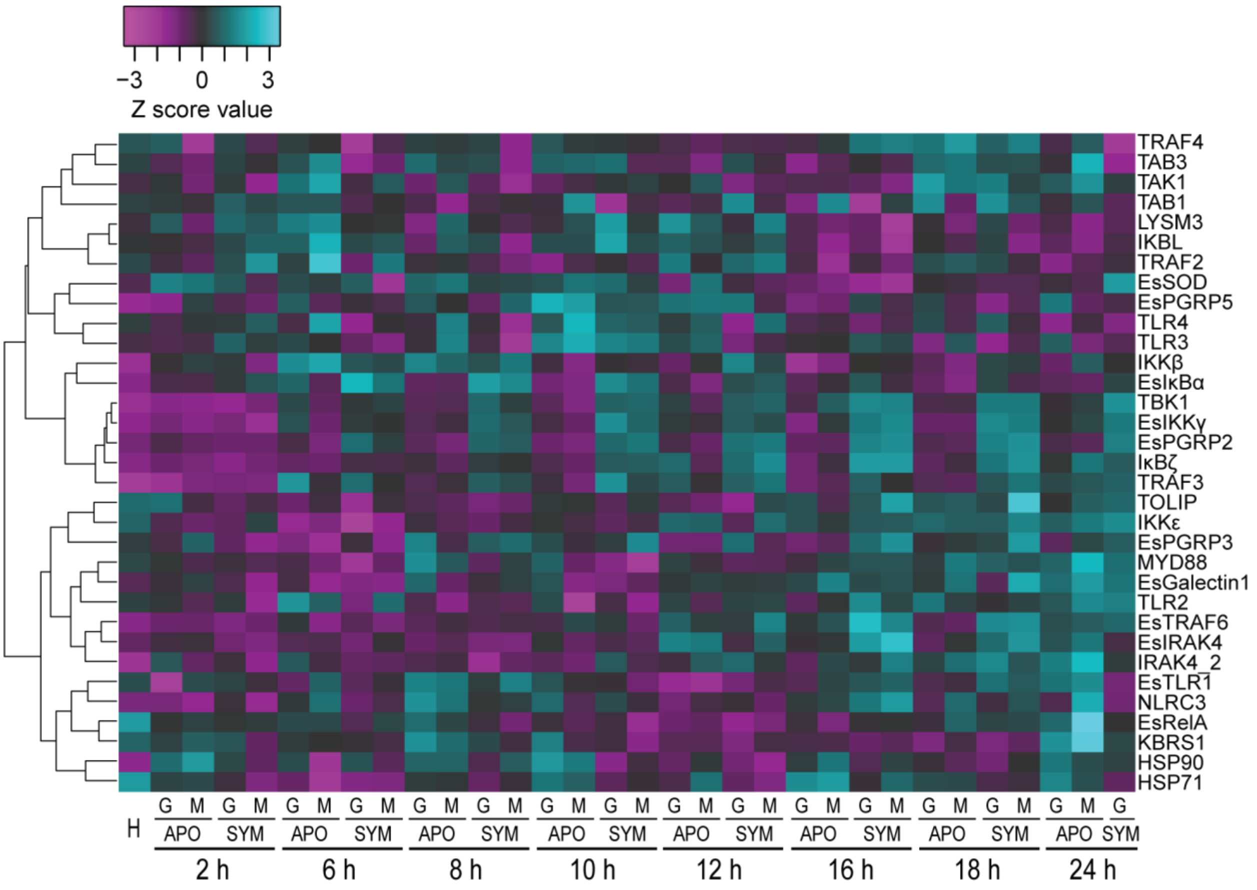

Figure S2. Heatmap of voom transformed (log-CPM) NanoString gene expression hierarchically clustered by dissimilarities. H, hatchling; G, gravity; M, low-shear modeled microgravity; APO, aposymbiotic; SYM, symbiotic. The teal color indicates upregulated expression whereas purple color represents downregulated expression in that associated condition.

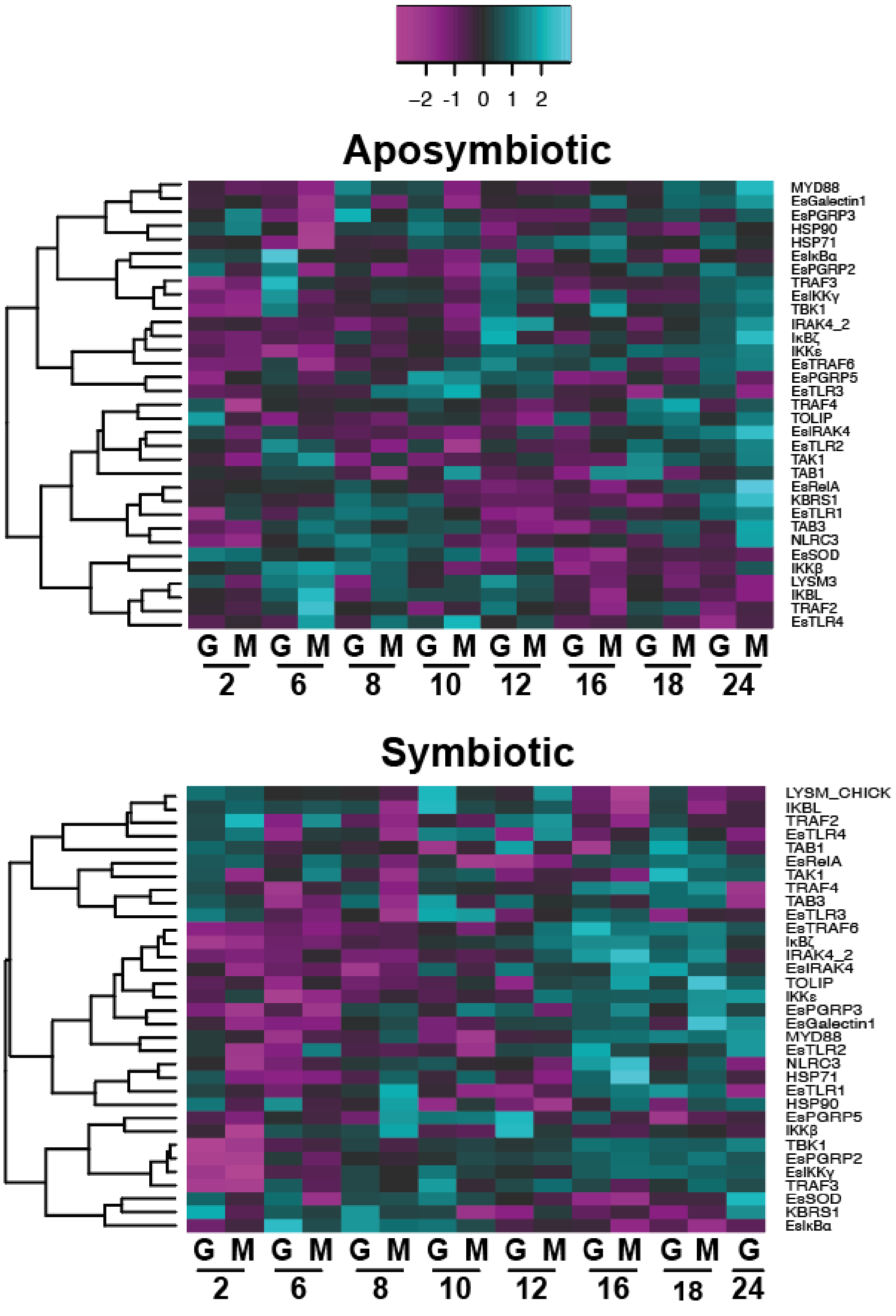

Figure S3. Heatmap of voom transformed (log-CPM) NanoString sample expression averaged by treatments and hierarchically clustered by dissimilarities of *V. fischeri*-infected light organs under gravity (G) and low-sheared modeled microgravity conditions (M) over time (hours). A detailed description of the targeted genes is listed in Table 1. The teal color indicates upregulated expression whereas purple color represents downregulated expression in that associated condition.

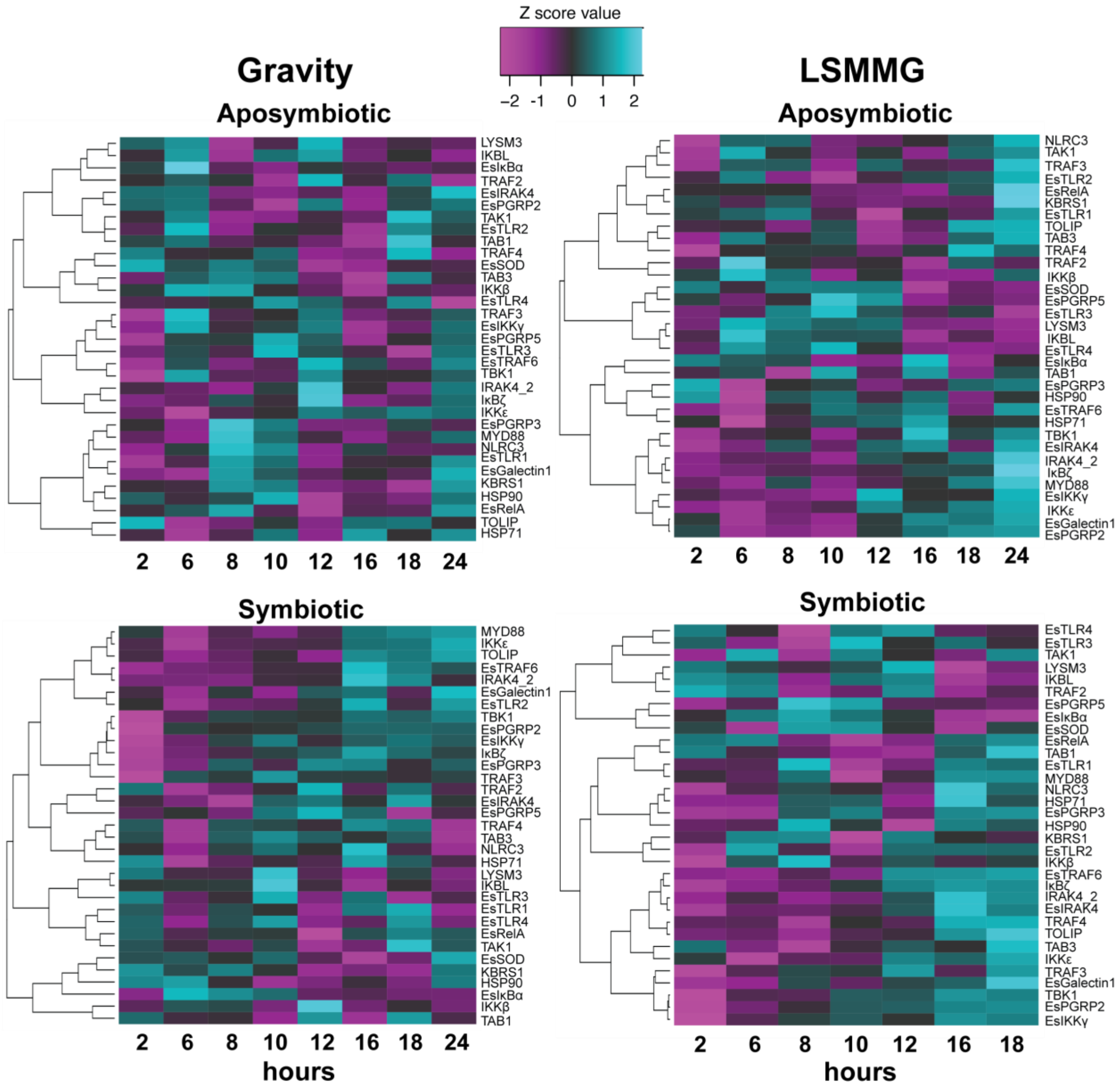

Figure S4. Heatmap of voom transformed (log-CPM) NanoString sample expression organized by gravity treatment and symbiosis state. Expression was averaged by treatments and hierarchically clustered by dissimilarities of *V. fischeri*-infected light organs under gravity and low-sheared modeled microgravity conditions (LSMMG) over time (hours). A detailed description of the targeted genes is listed in Table 1. The teal color indicates upregulated expression whereas purple color represents downregulated expression in that associated condition.

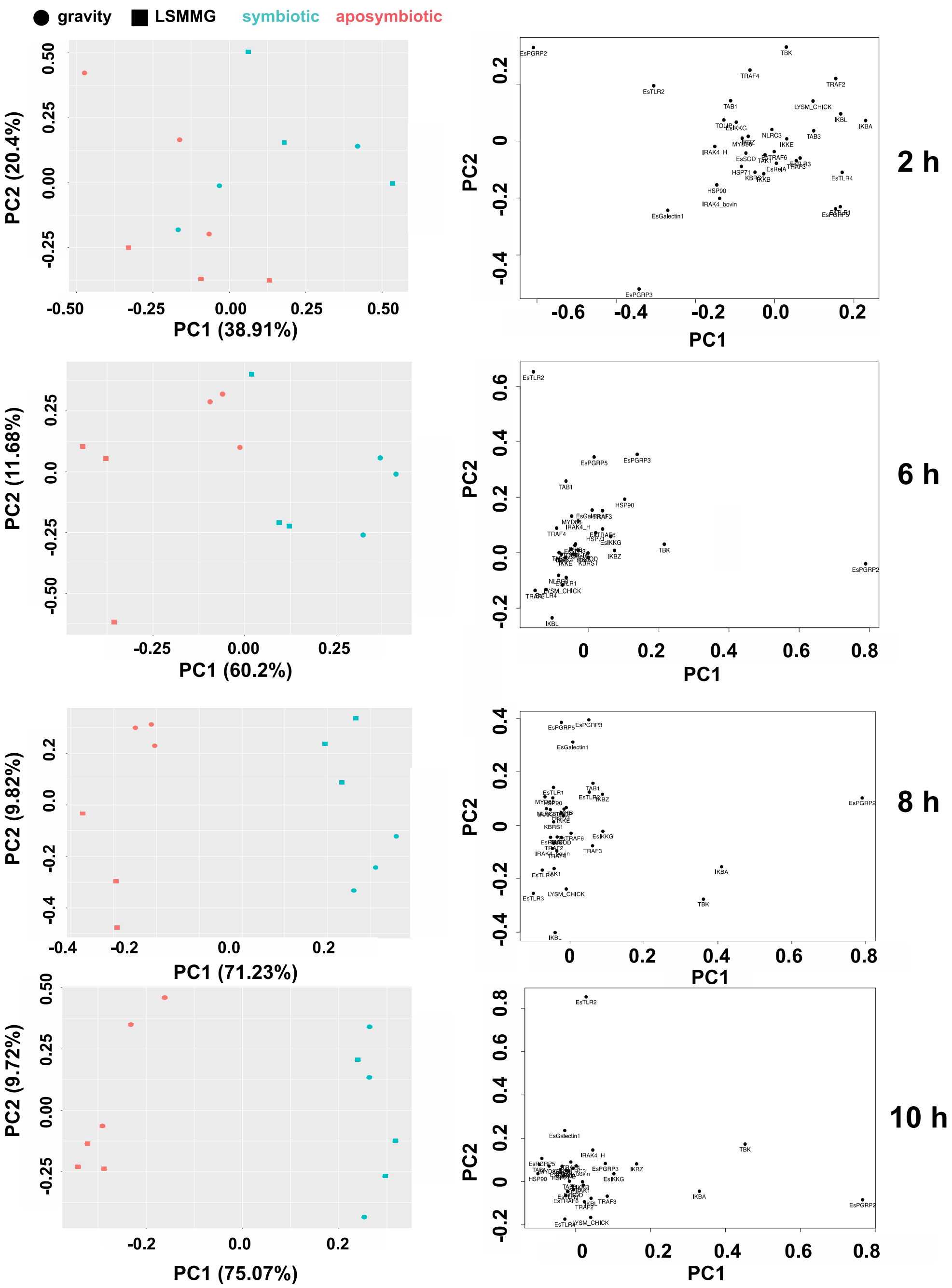

Figure S5. Principal component analysis (PCA) of all NanoString gene expression assay samples from 2 h - 10 h and their associated loading plots of genes driving the PCA under gravity (circle) and low-shear modeled microgravity (LSMMG, square) conditions in symbiotic (blue) and aposymbiotic (red) conditions.

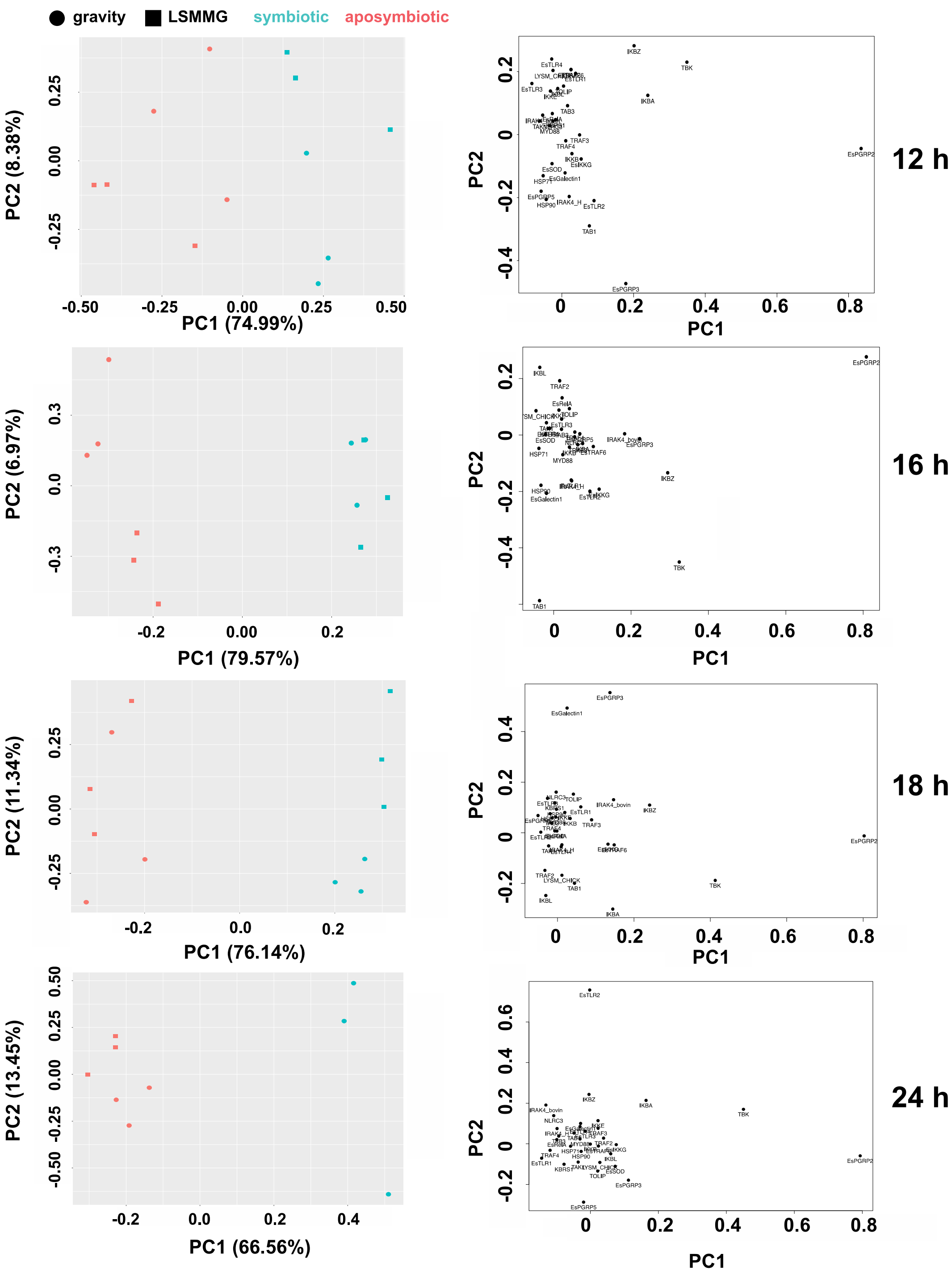

Figure S6. Principal component analysis (PCA) of all NanoString gene expression assay samples from 12 h - 24 h and their associated loading plots of genes driving the PCA under gravity (circle) and low-shear modeled microgravity (LSMMG, square) conditions in symbiotic (blue) and aposymbiotic (red) conditions.

Table S1. Targeted genes for NanoString expression assay probe design

Group	Gene name	Probe sequence (100bp; 5'- 3')
House-keeping	ACTB3	TCTATGAAGGTTATGCCCTTCCTCATGCCATTCTCCGTCTGGATTTGGCCGGTCGTG ACTTGACCGATTACCTCATGAAGATCCTCACGGAGCGTGGATA
	PYC1	AAGCCGCCTATGGAGGTGGTGGCCGTGGCATGCGGGTTGTCCGATCCTTGGATGA AGTAGCAGAGAATTTTGAAAGGGCGTATTCGGAAGCACTTTCTGC
PRR	EsPGRP1*	TATCCTTTCGTATGACCTCCTTCACGATCCCAACCACGACCTTTGTACACATAACCA TCTTCACCAACCAAGTAGTTGTAACCGAGATCCGACCATCCTC
	EsPGRP2	TTCATACACCACACAGCAATGGATTATTGCACCAACATCTCTACATGCTCCGAGCA AATGAGAAAAATCCAGAACTTTCATATGGACGACCGAGGATGGT
	EsPGRP3	CCCAACTACGAACTGTTTGGACATCGAGACGTTCGCAAAACAGAGTGTCCAGGAG AGAAGTTTTATCAATACATCAGAACATGGAAGCACTATAGCACTA
	EsPGRP4*	GATTTGTCATTGAAACCTCGGGTATGAGCGCCAACTCTGTCCCATCCTCGGCCTTC ATAAACCCGGCCATCTTCACCGATGATGAAATTATACCCAATAT
	EsPGRP5	CGATATTGGATATAACTTTCTGATCGGCAGCGACGGTAACGTGTACGAAGGACGT GGATCGGATACAGTTGGAGCCCACACCAAGTTCTACAATTCTCAG
	EsTLR1	TCCATTGGCCACTTGGCACCGAACGTGTTCTCATCTAACCGTCATCTCGAAGTGCT GATTCTAACCAACAATAGCTTGATTCACTTGGGTGAATACTCCC
	TLR2	ATCTTCAGATCACACCTGATACGATTGGAAGCCAACTGCCAAAGTTAAAGAAGTT ATACTTTATTGGAGTGCAGGACGTTTTACTAAGCGTGGACAACAA
	TLR3	ACAAGTCAGCTTGTCACATTCCGAACAGTTACAAGTATTGGTGTTAAGGCATGCTA AAATAAACATTCCAAAATTTGTGGAGCCGACGAATAATATGGCT
	TLR4	GTCGTCCTCCCGATGAACCTCAGCCGGACGCCTAACATGCAAGATCATTATGCCAA ATCGATCGGCATTGATGTCAAGGCTAATATGTTCCGCAGAATCC
	TLR6*	ATGCATTGTACGGTTTGAGAGAGACATCTCTGGAGACTCTGCATATCAATGCTATA CATAACATATTCGGTTTGGGAACAGCATTTACGGAAGAGGACGC
	EsGalectin1	TCTTCAGTCCGACTCTTCGGATGATGCTGTGATTGCTTTCCATCTGAACCCTCGATT GGATTCTAATGAAGTTTGCTGCAACACTTATGATGGCGGATGG
	LYSM2*	TTAATTGTTCAACCGACACGCCATATTTGAGTGCAATGCCGACCAAGGAATCCGTT GAGACAACTCTGTGCTTCACGAAATTAGAATTCCATCGAACAGG
	LYSM3	TATCACAGGTTACGGTTGAAGGATGACCAAGTCGAGAGGAAAGGAAGTCGACATC ACAATAACCAGCAACAGACTTATGTGTTTGGACACGTGGACACCG
Effector enzyme	EsSOD	ACTGTTGTGGTTCATGCAGATGTTGATGACTTGGGAAAAGGTGATCATGAGTTAAG CAAGACAACGGGTAATGCTGGTGGCCGACTTGCATGTGGAGTTA
	SODC2*	TTCGGCTTCCGGGTTGAGGAAAGAGAAATTCCCACAACCCTTGATTTCAGTGTAAT CATTTGATACCGCCATTGCTCGCTCCATCACGGATGAAGGACCA
	SODC3*	GACGACGACGGTGCCCATGTCTACTTGCACATGGACATGCACGACGATTTCGCCGA ACTTATTGAAGAGGGCGAATACGAACACTTCGGTCAACCATCAT
	HSP71	AAAGCGGTCGGGATCGATCTTGGAACCACCTACTCCTGTGTGGGTGTATTTCAACA TGGCAAGGTTGAAATTATCGCTAACGATCAGGGAAACCGAACAA

	HSP90	TCGAATGAAGGAAAACCAGAAAAATATCTATTACATAACTGGTGAGAGCCGAGAT GCTGTAAAGAATTCTGCTTTTGTAGAGAAAGTGACCAAATCTGGT
Signaling	MYD88	AACCTTGCCCAGTTGTTGGATGTTCCCTGTAAAGTCTTTGCCTCTGACTGGACCAGT TTGGGGGCGGAGGTTGGACTGACCTTTGTTGAAAAAATGTCAT
	EsIRAK4	ACGTGGTGGACAATCAACATTTTCTCATCAATGTACAGAACCTACAGAGCAGAGA AGTATTGACTCGTGTGATAGCTGTTGGAAGATATCTGACTCAAAG
	IRAK4_2	TATGGTAAATTAAAGGAAACTGACTGTGCCATAAAAAAACTGACCAAGGATTATT CTGAGGTAAATGCCGCCATGTGCAACCTGAAAAAGAATGCATTGG
	TRAF2	TTTGGTACCCTTTGCGAGGAAATCTGTCATCACTACTCCGTCCTGGTCCCAAAAGA CTCCGAGCATGACCTTGCTAGCGGAGGGCTGCACCCTTGCCTTC
	TRAF3	TAACACTTTGATTGAACAAATTTTGGAGCAGACCTCTGTGCATGACCGTGAGATTG GTGTCCACGATGTTAGATTTTCTGAGATGGAAGCCCGATTAGCA
	TRAF4	CAAGGAAGGCTGTAAATGGGTAGAATCACTCGCAAACTTACAGAGTCATTTAGAT GTTTGTCGTTATGATGCCGTCTCATGTCCCAACAGCTGCTCAGCT
	EsTRAF6	AGAAATTCTCTCCATGAAGGTGCGCTGTCCGAATAGAAAGTCTGGATGTAATACTT GTCTGGAGTTGAAACATATTGAGGAACACGCAGAAAGTTGTCCG
	TAK1	TCGGAAAAGGTGCCTTTGGAGTTGTCCGCCGTGCTATGTACCATGGGCATAGCGTT GCTGTAAAGTTGATTGAGACTGAAACTGAAAAGAAAGCCTTCAT
	TAB1	GCTGAGGGAATGGTAGAAATTTTTGCAAGTAGGGTGAAGTAGGAGTGCCACAAGT ATTGTCAGCATTAGCGACACCAAACAAGTAGTTAAAGTGACGCAC

Group	Gene name	Probe sequence (100bp; 5’- 3’)
	TAB3	CCCAAGTTCTGCCTCCTACTCGATGTTCAGCACACCACAGCTTTGCATCACTGGGC AGGTTCAACCGACCACATCTTCGCCTCAAACTTTACACGTTTCT
	IKKβ	GCATTTCAGCAATGGCTGAAGACAATGCTAATATGGGATAAAACGCTTCGCGGTA ACGATCATGAAGAAAAGGGCAAGAAACAAACGTGCTTCACCGAAC
	EsIKKγ	TTCAACTATTGAAAAGAAATACCACAGCGAAAAGAACCGAAATGATCAGCTGCAG GGTGCAATTGGTGAACATGCGTCATCTGAAAATGCAGTTGAGGTT
	IKKε	TACGAAGCTGTGTTCTTCAAAAGAGATTCCCAGAAGTTTGATGCCACTGCCGATTT ATGGAGTCTGGGTGCCACGCTTTATCACAGTGCAGCTGGCTTGC
	TBK1	AGGGAGACGGAAGAATAGAAAGTGTAGACTCGAAATGTTAAAATGGAAACCCTC AGGTGTGATATCTGCAATTGAAAATGAAAACGGGGAAATAATCTGG
	TOLIP	CCGACCTCTACTTACACCGAAGAGGATATTCAACTTGTGAAGGACATGTTTCCTGC CATCGAGACGGATGTGATCCAATCTGTTTTCATTGCACAACGAG
	EsRelA	CTGACCAGGCATGTAGTGAACCAAAAGAGTTTTTGTATCAGCCACAAGATCCAGA TCCTGATAGAATAGCAATAAAGCGAAAACGCAAAGCTTCAACTC
	NFκB1*	GACAGATTGAAACTTTTGGCGCCACGGATGTAATCATCAGCAATTTGTGC GTGTCCGCGAATAATAGCCAAATGCAATAAACTGGAAAGATAAAAATAA A
	EsIκBα	GTTATAGAAAAAAAAAAAATGCCATCACAACAGAAGAGCTGGCCGGACATGGGA GCAAACACGTCTTCAAACAATGAAGGAGTCTTTTTCCAGAGACACT

IκBζ	TCTGCCTTTGATGAGACCCATTTGAAAGACCTCTGTGAGATCATTGAGAAAGATCT CGTTAAAGAAAAGCTTGAAAAGGCGCTGTCTGAGGATTTGCCAC
IKBL1	ACGTGGGCTAATCGAATTTGGTCTGAGCATAAACGCAAGAGAGATTCTGGTGTATT TGCACGATGGAAAGAATCTATCCAACGGAAATGGCTGTTTGGGC
NLRC3	GCATTTTGCCAGGTGCTGCGGAACACAGACTCTTATCAGGATGTGTCTGAAATGCT GGACGCCATGACCAAAATCTCATTCATGATATCGGAGATCAAT
KBRS1	CGACAATCTCTTCAAGAACCTTTCGTTTGGCTCACCTCTCGGATAACCCAACCTCC AAGTAAATCCGCATTTCCCTTGGGAAGAAAGAATAAAGGAAACG

* Probes were unsuccessful in the NanoString assay
